# Low Levels of CD36 in Peripheral Blood Monocytes in Subclinical Atherosclerosis in Rheumatoid Arthritis: A Cross-Sectional Study in a Mexican Population

**DOI:** 10.1155/2014/736786

**Published:** 2014-06-09

**Authors:** Eduardo Gómez-Bañuelos, Beatriz Teresita Martín-Márquez, Erika Aurora Martínez-García, Mauricio Figueroa-Sanchez, Lourdes Nuñez-Atahualpa, Alberto Daniel Rocha-Muñoz, Pedro Ernesto Sánchez-Hernández, Rosa Elena Navarro-Hernandez, Perla Monserrat Madrigal-Ruiz, Adan Alberto Saldaña-Millan, Sergio Duran-Barragan, Laura Gonzalez-Lopez, Jorge Ivan Gamez-Nava, Mónica Vázquez-Del Mercado

**Affiliations:** ^1^Instituto de Investigación en Reumatología y del Sistema Musculo Esquelético, Centro Universitario de Ciencias de la Salud, Benemérita Universidad de Guadalajara, Sierra Mojada No. 950, Colonia Independencia, 44340 Guadalajara, JAL, Mexico; ^2^Departamento de Fisiología, Centro Universitario de Ciencias de la Salud, Benemérita Universidad de Guadalajara, Sierra Mojada No. 950, Colonia Independencia, 44340 Guadalajara, JAL, Mexico; ^3^Servivio de Radiología e Imagen, Hospital Civil de Guadalajara “Fray Antonio Alcalde”, Benemérita Universidad de Guadalajara, Hospital No. 278, 44280 Guadalajara, JAL, Mexico; ^4^Laboratorio de Inmunología, Departamento de Fisiología, Centro Universitario de Ciencias de la Salud, Benemérita Universidad de Guadalajara, Sierra Mojada No. 950, Colonia Independencia, 44340 Guadalajara, JAL, Mexico; ^5^Departamento de Medicina Interna-Reumatología, Hospital General Regional No. 110, Instituto Mexicano del Seguro Social, Circunvalación Oblatos No. 2212, Colonia Oblatos, 44700 Guadalajara, JAL, Mexico; ^6^Unidad Médica de Alta Especialidad, Centro Médico Nacional de Occidente, Instituto Mexicano del Seguro Social, Belisario Domínguez No. 1000, Independencia Oriente, 44340 Guadalajara, JAL, Mexico; ^7^Servicio de Reumatología, Hospital Civil “Dr. Juan I. Menchaca”, Benemérita Universidad de Guadalajara, Salvador de Quevedo No. 750, 44100 Guadalajara, JAL, Mexico

## Abstract

Patients with rheumatoid arthritis (RA) have a higher risk for atherosclerosis. There is no clinical information about scavenger receptor CD36 and the development of subclinical atherosclerosis in patients with RA. The aim of this study was to evaluate the association between membrane expression of CD36 in peripheral blood mononuclear cells (PBMC) and carotid intima-media thickness (cIMT) in patients with RA. *Methods*. We included 67 patients with RA from the Rheumatology Department of Hospital Civil “Dr. Juan I. Menchaca,” Guadalajara, Jalisco, Mexico. We evaluated the cIMT, considering subclinical atherosclerosis when >0.6 mm. Since our main objective was to associate the membrane expression of CD36 with subclinical atherosclerosis, other molecules related with cardiovascular risk such as ox-LDL, IL-6, and TNF*α* were tested. *Results*. We found low CD36 membrane expression in PBMC from RA patients with subclinical atherosclerosis (*P* < 0.001). CD36 mean fluorescence intensity had negative correlations with cIMT (*r* = −0.578, *P* < 0.001), ox-LDL (*r* = −0.427, *P* = 0.05), TNF*α* (*r* = −0.729, *P* < 0.001), and IL-6 (*r* = −0.822, *P* < 0.001). *Conclusion*. RA patients with subclinical atherosclerosis showed low membrane expression of CD36 in PBMC and increased serum proinflammatory cytokines. Further studies are needed to clarify the regulation of CD36 in RA.

## 1. Introduction 

Rheumatoid arthritis (RA) is a chronic autoimmune disease with systemic complications and early cardiovascular death [1]. RA patients are prone to develop atherosclerosis at a relatively young age.

Atherosclerosis and inflammation in RA share several mechanisms in their pathogenesis including proinflammatory cytokine expression, infectious agents, dyslipidemia, and autoantibodies [2–8].

Type B scavenger receptors (SR), like CD36, are molecules possibly involved in the pathogenesis of atherosclerosis. During atherogenesis, blood monocytes are recruited into the intima and subintima layers of blood vessels; were they internalize oxidized low density lipoproteins (ox-LDL) through SR (CD36). This process results in the activation of monocytes and their differentiation into macrophages and foam cells. As a consequence, matrix metalloproteinases, proinflammatory cytokines, and chemoattractants enhance inflammatory infiltrates and vascular remodeling [9, 10]. CD36 has a critical role in the atherosclerotic plaque development [11–14]. However, their role in cardiovascular complications of RA has not been studied.

The aim of this study was to evaluate the association between membrane expressions of CD36 in peripheral blood mononuclear cells (PBMC) with carotid intima-media thickness (cIMT) in patients with RA without known traditional cardiovascular risk factors.

## 2. Methods

### 2.1. Patients

We recruited RA patients that met ACR 1987 criteria [15], from the Hospital Civil “Dr. Juan I. Menchaca” at Guadalajara, Jalisco, Mexico. Patients with known cardiovascular risk factors such as history of myocardiopathy, hypertension, diabetes mellitus, hyperlipidemia, malignancy, thyroid, renal or hepatic disease, smokers and steroid treatment >10 mg/day were excluded.

A structured questionnaire was applied to each patient to evaluate demographical and clinical variables. Physical examination, joint assessment, and venous blood drawn were performed at the visit.

### 2.2. Clinical Assessment

Disease activity was evaluated using Disease Activity Score 28 (DAS28) and C-reactive protein (CRP).

### 2.3. cIMT

It was assessed according to the recommendations defined by the Mannheim carotid intima-media thickness and plaque consensus (2004–2006–2011) [16] by a single operator using a high-resolution B-mode ultrasound (Philips Saronno, Italy) with a 9 MHz transducer. Two segments from the common carotid artery (CCA), one from the carotid bifurcation (BF), and two from the internal carotid artery (ICA) were evaluated. Mean cIMT values were calculated for each segment. Patients were classified according to the cIMT with a cut-off point of 0.6 mm.

### 2.4. Laboratory Assessment

Serum was obtained by centrifugation of whole blood at 2,000 rpm for 15 minutes; aliquots with serum were stored at −70°C for no longer than 6 months. Erythrocyte sedimentation rate (ESR) was measured using Wintrobe method and CRP by immunoturbidimetry (assay range 0.3–161 mg/L, Randox laboratories limited); total cholesterol (TC), triglycerides (Tg), high density lipoprotein cholesterol (HDL-c), and low density lipoprotein cholesterol (LDL-c) were measured by routine methods. Cardiovascular risk ratio was calculated using the atherogenic index of plasma (AIP) which was defined as TC/HDL-c. Anticyclic citrullinated peptide (CCP) antibodies (intra-assay variation coefficient (VC) < 9% and interassay VC < 11%, Axis-Shield Diagnostics Ltd.), serum interleukin (IL)-6 (intra-assay VC 5.1%–7.7% and interassay VC 6.5%–9.3%, Invitrogen), tumor necrosis factor (TNF)*α* (intra-assay VC 4.2%–5.2% and interassay VC 4.6%–7.4%, R&D Systems), and ox-LDL (intra-assay VC 3.9%–5.7% and interassay VC 9.0%–11.0%, ALPCO Diagnostics) were measured by enzyme-linked immunosorbent assay (ELISA).

The flow cytometric analysis was performed using fluorescein isothiocyanate- (FITC-) conjugated mouse monoclonal antibodies against human CD36 and PE conjugated anti-human CD14 (BioLegend). PBMC were obtained by density gradient centrifugation using a lymphocyte separation solution. The cells were washed twice with phosphate buffered saline (PBS) and fixed with 1% paraformaldehyde for 20 minutes at 4°C. After being washed with PBS, 5 × 10^6^ cells in 50 *μ*L PBS were incubated with FITC or PE-conjugated monoclonal antibodies for 30 minutes at 4°C. The cells were then washed twice before being assayed with a flow cytometer (Beckman Coulter, Epic XL, Miami, FL, USA) and analyzed with the software WinMDI 2.9.

### 2.5. Statistical Analyses

Values are presented as mean ± standard deviation (SD) and percentages as appropriate. Between-group differences were estimated by independent-sample Student's *t*-test. Chi-square test (or Fisher's exact test) was used to compare categorical variables. Spearman's correlation coefficient was calculated for cIMT, DAS28, CRP, anti-CCP, IL-6, and TNF*α*. All data were analyzed using SPSS 18.0 software (SPSS Inc., Chicago, IL) and replicated using the software Stata 12.0 (StataCorp LP, Texas, USA), considering a two-tailed level of *P* < 0.05 statistically significant.

### 2.6. Ethics

Protocol was approved by the IRB committee (register number 1068/10) of the Hospital Civil “Dr. Juan I. Menchaca” of the Benemérita Universidad de Guadalajara.

## 3. Results

Sixty-seven patients were included in this study; 60 (89.5%) were female, with a mean (SD) age of 44.2 (11.9) years old; 29 (43.28%) had evidence of increased cIMT.  Table 1 shows the comparison of RA subgroups with and without increased cIMT. No statistical differences in age, disease duration, and disease activity were observed between higher and lower cIMT groups. The increased cIMT group (>0.6 mm) showed higher serum levels of TC (*P* < 0.001), Tg (*P* = 0.002), ox-LDL (*P* < 0.001), and AIP (*P* < 0.001) and lower serum levels of HDL-c (*P* < 0.001) compared with the cIMT group (<0.6 mm). Serum levels of CRP, TNF*α*, IL-6, and anti-CCP also were higher in the increased cIMT group (*P* < 0.001).

### 3.1. CD36 PBMC Membrane Expression

RA patients with increased cIMT showed lower levels of CD36 compared with no increased cIMT (67.09 ± 27.50 versus 170.43 ± 38.80, *P* < 0.001).

The PBMC membrane expression of CD36 MFI was significantly lower in patients with moderate and high disease activity (*n* = 22, 64.31 ± 16.72), when compared to patients with low disease activity (*n* = 11, 129.78 ± 13.73) or in remission (*n* = 34, 158.2 ± 13.66) (*P* < 0.05).

### 3.2. Correlations Coefficients between cIMT, Clinical, and Laboratory Characteristics of RA Patients

Correlation coefficients between cIMT and characteristics of RA patients are shown in Table 2. cIMT was negatively correlated with CD36 MFI and HDL-c and positively correlated with age, TC, Tg, AIP, anti-CCP, TNF*α*, IL-6, CRP, and ox-LDL.

**Figure 1:**
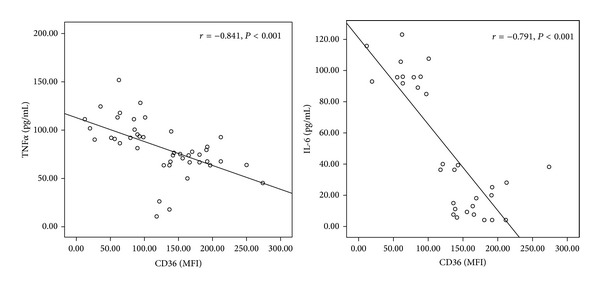
Correlation between serum TNF*α*, IL-6, and CD36 MFI.

Figure 1 showed a negative correlation between CD36 MFI with TNF*α* (*r* = −0.729, *P* < 0.001) and IL-6 (*r* = −0.822, *P* < 0.001). In data not shown, we observed a negative correlation of CD36 MFI with ox-LDL (*r* = −0.841, *P* < 0.001).

## 4. Discussion

In this study, we showed that RA patients with subclinical atherosclerosis showed low membrane expression of CD36 in PBMC and increased serum proinflammatory cytokines (Table 1). The CD36 PBMC membrane expression was negatively correlated with cIMT, ox-LDL, TNF*α*, and IL-6 (data not shown). We described a positive correlation between age, TC, Tg, ox-LDL, AIP, CRP, TNF*α*, IL-6, and anti-CCP antibodies with cIMT (Table 2).

In endothelial cell cultures exposed to IL-6 and TNF*α*, upregulation of the scavengers receptors- (SR-) A and ox-LDL receptor- (LOX-) 1 was shown but not CD36 expression. Endothelial cells stimulated with human sera rich in IL-6 and TNF*α* from RA patients; the CD36 expression increased and was not modified by IL-6 or TNF*α* antagonists. This suggests that a different factor present in the serum of these patients, like ox-LDL, may be responsible for the upregulation of CD36 [17].

TNF*α* promotes atherosclerosis through the inhibition of cholesterol efflux, favoring the cholesterol uptake by CD36 and other SR via protein kinase pathway. In THP-1 cells in the presence of ox-LDL, TNF*α* impaired the cholesterol efflux by downregulation of ATP-binding cassette (ABCA) proteins [18].

Boyer et al. showed the downregulation of membrane expression and mRNA levels of CD36 in culture of fresh PBMC from healthy donors in the presence of human recombinant TNF*α*. In other experiment incubating PBMC with a humanized TNF*α* blocker (Adalimumab), the membrane and mRNA CD36 increased [19]. The authors concluded that different pathways were involved in the regulation of CD36. When TNF*α* was used, the signaling was mediated by a reduction in activated peroxisome proliferator-activated receptor gamma, whereas Adalimumab increased CD36 through redox signaling.

In our report, the membrane CD36 in PBMC was decreased in RA patients with higher cIMT; besides, a negative correlation between TNF*α* and membrane CD36 MFI was found. These support the findings observed in endothelial cells and PMBC cultures reported by Boyer et al. [19]. However, these results must be corroborated by further studies using similar approaches as described before.

In our patients, another possible explanation for the low levels of CD36 might be the proteolytical cleavage by ADAM17, which might result in more soluble CD36 [20]. It has been reported the protective role of the CD36 polymorphism, G573A, in plaque thickness in patients with early coronary artery disease [21].

In a more detailed analysis of our results, we looked for the influence of disease duration and treatment. We found that, RA patients with normal cIMT had longer disease duration and lower levels of TNF*α* and IL-6 (Table 1) probably due to the benefit of prolonged use of antirheumatic drugs in the prevention of subclinical atherosclerosis [22].* In vitro* studies using methotrexate (MTX) favor the cholesterol efflux through activation of adenosine A2 receptor, which in turn prevents the foam cell differentiation and atherosclerosis plaque formation [23, 24]. MTX might downregulate serum TNF*α* in RA [25]. A large study that enrolled more than 8,000 patients using synthetic DMARDS compared with anti-TNF*α* users (11,000 approximately) showed a reduction in cardiovascular risk in both groups, even though the reduction was greater in the anti-TNF*α* treated patients [26, 27].

Based on our results, low PBMC CD36 membrane expression showed a negative correlation with cIMT, ox-LDL, TNF*α*, IL-6, and DAS28. From the clinical standpoint, the interaction between these factors might reflect the importance of CD36 in the development of atherosclerosis in RA.

## 5. Conclusion

RA patients with subclinical atherosclerosis showed low membrane expression of CD36 in PBMC and increased serum proinflammatory cytokines. Translation of the results from these studies to the clinical field is difficult since the functional role of CD36 depends on the target cell. Further studies are needed to validate our findings and clarify the downregulation of CD36 in RA.

## Figures and Tables

**Table 1:** Characteristics and comparison of RA subgroups with and without increased cIMT.

Variable	Study groups		*P*
	cIMT ≤ 0.6 mm *n* = 38	cIMT > 0.6 mm *n* = 29	
Age, years	42.58 ± 11.43	47.74 ± 12.54	0.14
*RA characteristics *			
Disease duration, years	4.52 ± 4.46	3.40 ± 5.50	0.47
DAS28, units	2.73 ± 0.98	3.48 ± 1.12	0.03
*Lipid profile *			
TC, mg/dL	176.39 ± 34.83	239.78 ± 44.31	<0.001
Tg, mg/dL	136.87 ± 58.22	195.33 ± 63.95	0.002
HDL-c, mg/dL	51.87 ± 15.34	36.74 ± 8.40	<0.001
LDL-c, mg/dL	109.00 ± 24.50	111.45 ± 27.74	0.75
VLDL-c, mg/dL	27.79 ± 10.91	32.85 ± 16.10	0.17
ox-LDL, mg/dL	55.62 ± 5.38	219.48 ± 98.58	<0.001
AIP, TC/HDL-c	3.66 ± 1.26	6.77 ± 1.72	<0.001
*Serological profile *			
ESR, mm/h	24.03 ± 19.67	21.14 ± 9.03	0.52
RF, IU/mL	97.18 ± 101.12	134.18 ± 133.94	0.61
CRP, mg/L	3.83 ± 2.61	13.29 ± 6.31	<0.001
TNF*α*, pg/mL	64.72 ± 9.28	104.75 ± 17.49	<0.001
IL-6, pg/mL	29.03 ± 3.43	99.45 ± 11.29	<0.001
Anti-CCP, U/mL	73.22 ± 65.92	154.62 ± 97.70	0.004
*Flow cytometry *			
CD36, MFI	170.43 ± 38.80	67.09 ± 27.50	<0.001
*DMARDs *			
Methotrexate, *n* (%)	36 (94.7)	29 (100)	0.14
Time of use, years	4.51 ± 4.42	3.30 ± 5.27	0.06
Chloroquine, *n* (%)	21 (55.3)	15 (51.7)	1.00
Sulfasalazine, *n* (%)	9 (23.7)	4 (13.8)	0.52
Azathioprine, *n* (%)	6 (15.8)	4 (13.8)	1.00
Corticosteroids, *n* (%)	3 (7.9)	1 (3.5)	0.45

RA: rheumatoid arthritis; cIMT: carotid intima-media thickness; DAS28: disease activity score; TC: total cholesterol; Tg: triglycerides; HDL-c: high density lipoprotein cholesterol; LDL-c: low density lipoprotein cholesterol; VLDL-c: very low density lipoprotein cholesterol; AIP: atherogenic index of plasma; ESR: erythrocyte sedimentation rate; RF: rheumatoid factor; CRP: C-reactive protein; TNF*α*: tumor necrosis factor alpha; IL: interleukin; anti-CCP: anticyclic citrullinated peptides; MFI: mean fluorescence intensity; DMARDs: disease-modifying antirheumatic drugs.

Qualitative variables are expressed as frequencies (%); quantitative variables are expressed as means ± standard deviations (SD). Comparisons between proportions were computed using Chi-square or Fisher exact test. Comparisons between medians were computed with unpaired Student's *t*-test.

**Table 2:** Correlation coefficients between cIMT and characteristics of the groups evaluated.

Baseline variable	cIMT (mm)	
	*r*	*P*
Age, years	0.564	<0.001
Disease duration, years	−0.063	0.65
DAS28, units	0.159	0.26
TC, mg/dL	0.331	0.03
Tg, mg/dL	0.393	0.009
HDL-c, mg/dL	−0.316	0.04
LDL-c, mg/dL	0.285	0.06
VLDL-c, mg/dL	0.270	0.07
ox-LDL, mg/dL	0.457	0.007
AIP, TC/HDL-c	0.687	0.01
ESR, mm/h	−0.180	0.24
RF, IU/mL	−0.001	0.99
CRP, mg/L	0.579	0.001
TNF*α*, pg/mL	0.552	0.002
IL-6, pg/mL	0.681	<0.001
Anti-CCP, U/mL	0.393	0.05
CD36	−0.578	<0.001

cIMT: carotid intima-media thickness; RA: rheumatoid arthritis; DAS28: disease activity score; TC: total cholesterol; Tg: triglycerides; HDL-c: high density lipoprotein cholesterol; LDL-c: low density lipoprotein cholesterol; VLDL-c: very low density lipoprotein cholesterol; AIP: atherogenic index of plasma; ESR: erythrocyte sedimentation rate; RF: rheumatoid factor; CRP: C-reactive protein; TNF*α*: tumor necrosis factor alpha; IL-6: interleukin 6; anti-CCP: anticyclic citrullinated peptide antibodies; MFI: mean fluorescence intensity. Spearman *r* test.
